# Glioblastoma multiforme influence on the elemental homeostasis of the distant organs: the results of inter-comparison study carried out with TXRF method

**DOI:** 10.1038/s41598-024-51731-2

**Published:** 2024-01-13

**Authors:** Aleksandra Wilk, Zuzanna Setkowicz, Dariusz Banas, Ramón Fernández-Ruiz, Eva Marguí, Katarzyna Matusiak, Pawel Wrobel, Jolanta Wudarczyk-Mocko, Natalia Janik-Olchawa, Joanna Chwiej

**Affiliations:** 1 Faculty of Physics and Applied Computer Science, AGH University of Krakow, Krakow, Poland; 2 Institute of Zoology and Biomedical Research, Jagiellonian University, Krakow, Poland; 3 Institute of Physics, Jan Kochanowski University, Kielce, Poland; 4 Holy Cross Cancer Center, Kielce, Poland; 5 Interdepartmental Research Service (SIdI), Autonomous University of Madrid, Madrid, Spain; 6 Department of Chemistry, University of Girona, Girona, Spain

**Keywords:** Biophysics, Cancer, Chemical biology, Biomarkers, Diseases, Medical research, Chemistry, Physics

## Abstract

Glioblastoma (GBM) is a fast-growing and aggressive brain tumor which invades the nearby brain tissue but generally does not spread to the distant organs. Nonetheless, if untreated, GBM can result in patient death in time even less than few months from the diagnosis. The influence of the tumor progress on organs other than brain is obvious but still not well described. Therefore, we examined the elemental abnormalities appearing in selected body organs (kidney, heart, spleen, lung) in two rat models of GBM. The animals used for the study were subjected to the implantation of human GBM cell lines (U87MG and T98G) characterized by different levels of invasiveness. The elemental analysis of digested organ samples was carried out using the total reflection X-ray fluorescence (TXRF) method, independently, in three European laboratories utilizing various commercially available TXRF spectrometers. The comparison of the data obtained for animals subjected to T98G and U87MG cells implantation showed a number of elemental anomalies in the examined organs. What is more, the abnormalities were found for rats even if neoplastic tumor did not develop in their brains. The most of alterations for both experimental groups were noted in the spleen and lungs, with the direction of the found element changes in these organs being the opposite. The observed disorders of element homeostasis may result from many processes occurring in the animal body as a result of implantation of cancer cells or the development of GBM, including inflammation, anemia of chronic disease or changes in iron metabolism. Tumor induced changes in organ elemental composition detected in cooperating laboratories were usually in a good agreement. In case of elements with higher atomic numbers (Fe, Cu, Zn and Se), 88% of the results were classified as fully compliant. Some discrepancies between the laboratories were found for lighter elements (P, S, K and Ca). However, also in this case, the obtained results fulfilled the requirements of full (the results from three laboratories were in agreement) or partial agreement (the results from two laboratories were in agreement).

## Introduction

The total reflection X-ray fluorescence (TXRF) spectroscopy is a variant of conventional energy dispersive X-ray fluorescence and belongs to the instrumental methods of multi-elemental analysis. The source-sample-detector geometry used in TXRF method leads to significant decrease of the recorded background and simultaneous increase of the intensity of atomic fluorescence signals^1–4^. Numerous advantages of TXRF, including small amount of sample required for the measurement, wide range of examined concentrations, low detection limits, simple quantification based on internal standard method as well as cost-effectiveness cause that, for a few decades, the method has been successfully used in many fields of science and technique, among others in biomedicine, pharmacy, environmental sciences or mining and fuel industry^5–14^.

Better understanding of the physical processes underlying the generation of TXRF spectra, the use of modern technological solutions in the field of X-ray generation and detection and new/better methods of data recording and analysis as well as the improvement of practical aspects of measurements, made the method highly reliable, easy to use and user-friendly^1,3,15–20^.

*Glioblastoma multiforme* (GBM) is one of the most malignant human tumors overall^21,22^. As the early symptoms of GBM are not specific, it is usually diagnosed in its final stages. Even after introducing an appropriate treatment regimen, the prognosis is poor and the survival rate of patients is very low. According to WHO, the patients suffering from GBM live from 9 to 30 months from diagnosis^21,23^. The high malignancy of GBM, restricted treatment possibilities and poor prognosis push the medicine doctors and scientists to search for markers characteristic for early stages of disease development and more effective methods of its diagnosis and therapy^22–27^.

The mechanisms of cancerogenesis have been tested for decades. Despite this, the gathered knowledge allows only to some degree understand the pathogenesis of neoplasm diseases. The exact etiology of GBM is still not well known but among its risk factors the genetic predispositions (diseases that increase susceptibility to cancer, such as neurofibromatosis and Li-Fraumeni syndrome, as well as previous radiotherapeutic cycles), harmful environmental factors (long-term exposure to pesticides, smoking, working in oil refining or rubber production) and lifestyle (eating processed meat, exposure to formaldehyde) are taken into account^24,27,28^.

GBM can develop from healthy glial cells, as well as from an early-stage astrocytoma fibrillare. It is suspected that oligodendrocytes and stem cells may also transform into GBM. Almost all glioblastomas take the form of a tumor, which during its growth has the form of foci of necrosis surrounded by a layer of anaplastic cancer cells with hyperplastic blood vessels. Such complicated morphology causes that the complete surgical resection of tumor is very difficult and after the surgery GBM usually recurs^21,22^.

Determining the influence of the GBM development within brain on the whole organism has been still a great challenge. Stages and mechanisms of aggressive expansion of neoplastic cells into their surroundings, as well as genetic indicators of the cancerogenesis itself are known to medicine. However, besides the process of metastasis, the influence of GBM on other than brain vital organs is still a matter of speculation and the information on the elemental abnormalities occurring in the distant organs may shed some new light on this problem^29–33^. Therefore, the purposes of our study was determination of the changes in the concentrations of major, minor and trace elements which occur in kidney, heart, spleen and lung in rats subjected to implantation of human GBM cells to the brain. For the multi-elemental analysis of tissues the TXRF method was applied and the measurements of digested organ samples were done, independently, in 3 European laboratories. The data obtained there were compared to verify how not fully satisfactory values of validation parameters obtained during our previous inter-comparison research^34^, especially for light elements, may influence the results and subsequent conclusions from the real experiment.

## Results

**Figure 1:**
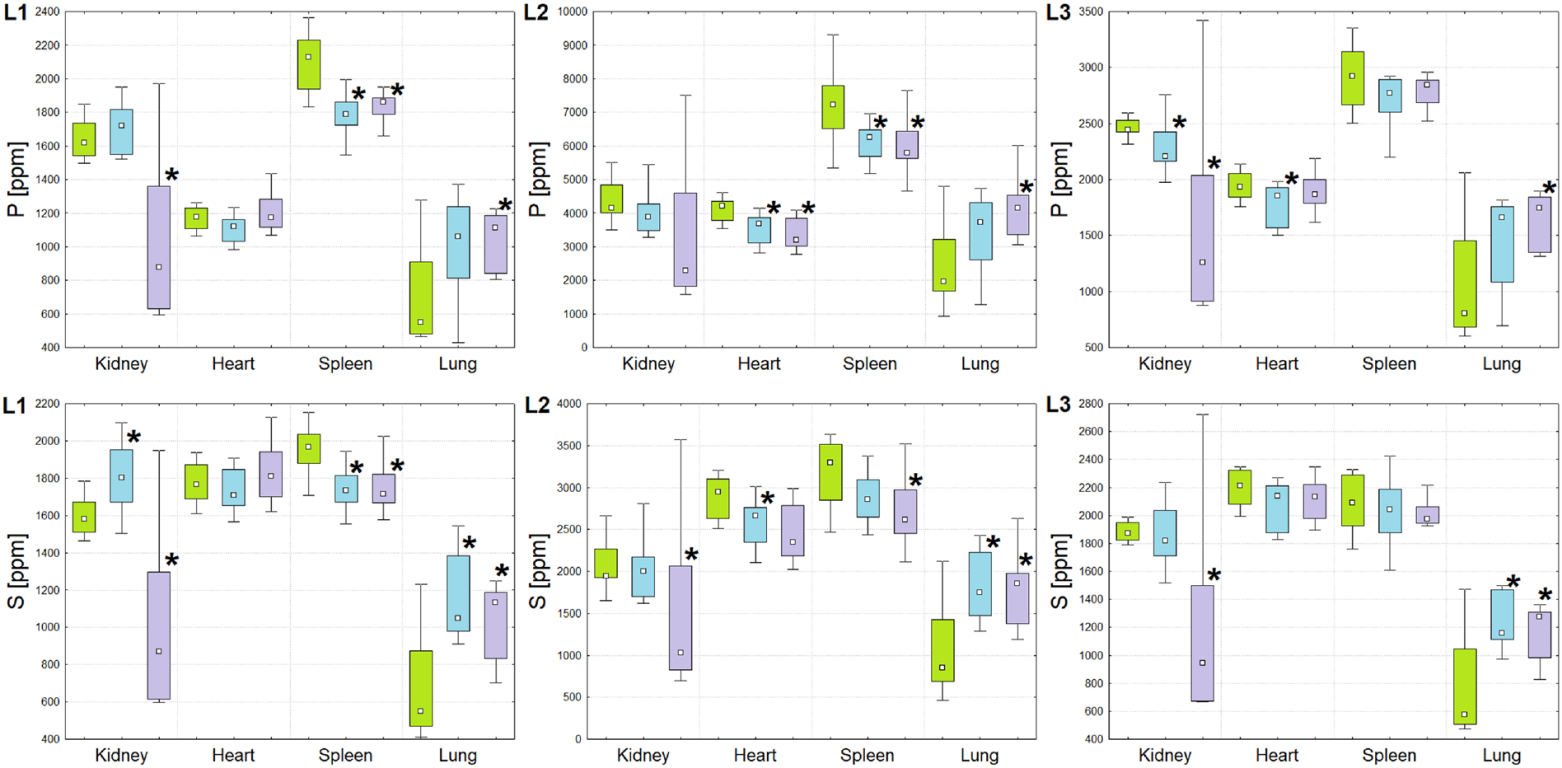
Box-and-whiskers plots presenting the ranges of P and S concentrations in kidneys, hearts, spleens and lungs taken from animals representing N (green), T (blue) and U (violet) groups obtained through the cooperating laboratories (from left to right: L1, L2 and L3). Median, interquartile range and minimal-maximal values are marked as a small square, a box and whiskers, respectively. The statistically significant differences (*p*-value < 0.05), comparing to control group, are signed with stars.

**Figure 2:**
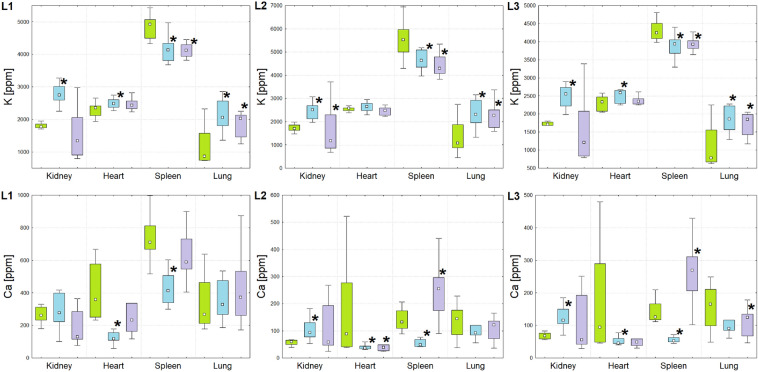
Box-and-whiskers plots presenting the ranges of K and Ca concentrations in kidneys, hearts, spleens and lungs taken from animals representing N (green), T (blue) and U (violet) groups obtained through the cooperating laboratories (from left to right: L1, L2 and L3). Median, interquartile range and minimal-maximal values are marked as a small square, a box and whiskers, respectively. The statistically significant differences (*p*-value < 0.05), comparing to control group, are signed with stars.

**Figure 3:**
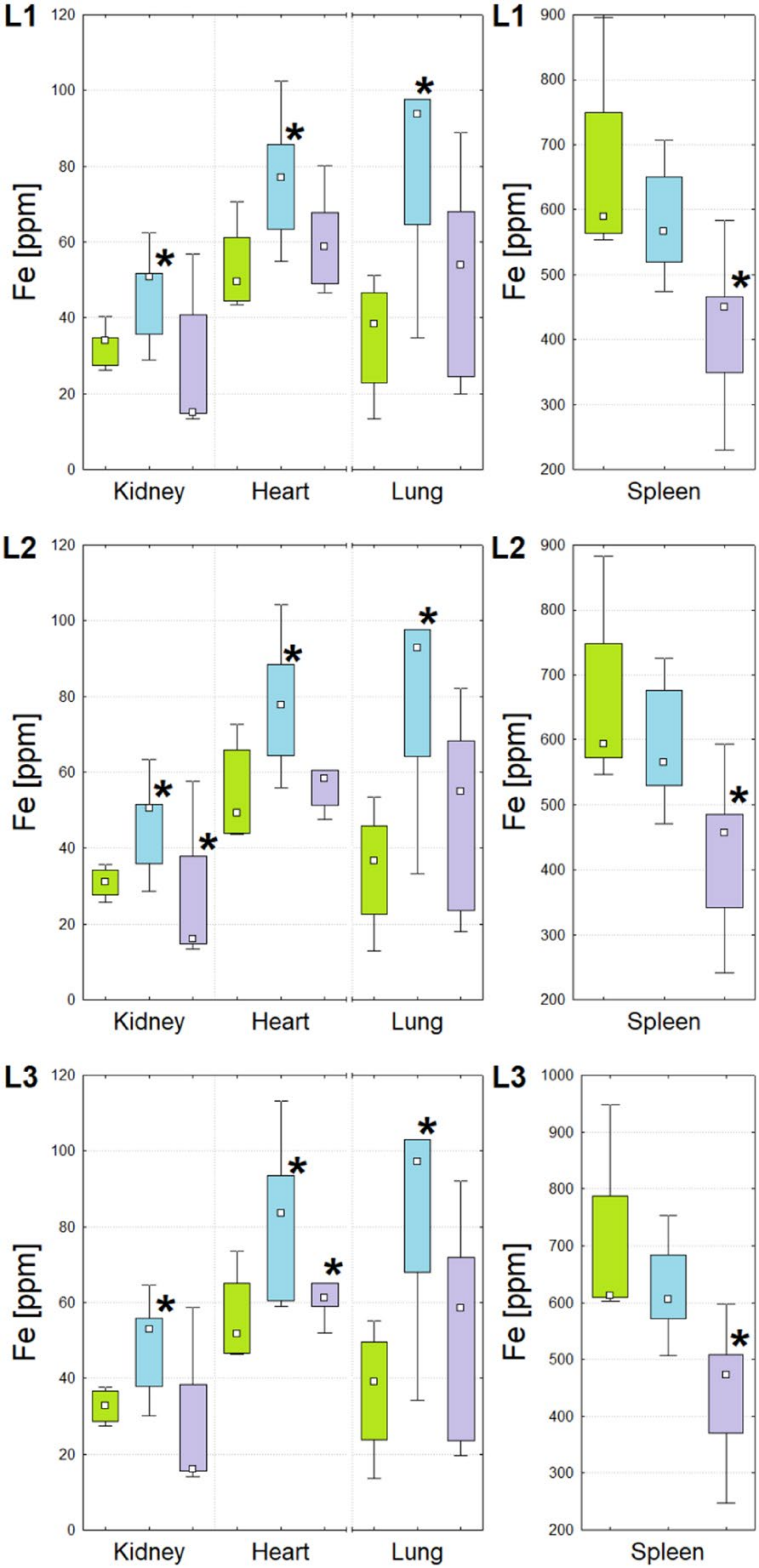
Box-and-whiskers plots presenting the ranges of Fe concentration in kidneys, hearts, lungs and spleens taken from animals representing N (green), T (blue) and U (violet) groups obtained through the cooperating laboratories. Median, interquartile range and minimal-maximal values are marked as a small square, a box and whiskers, respectively. The statistically significant differences (*p*-value < 0.05), comparing to control group, are signed with stars. For better visualization the data obtained for spleen were presented separately.

**Figure 4:**
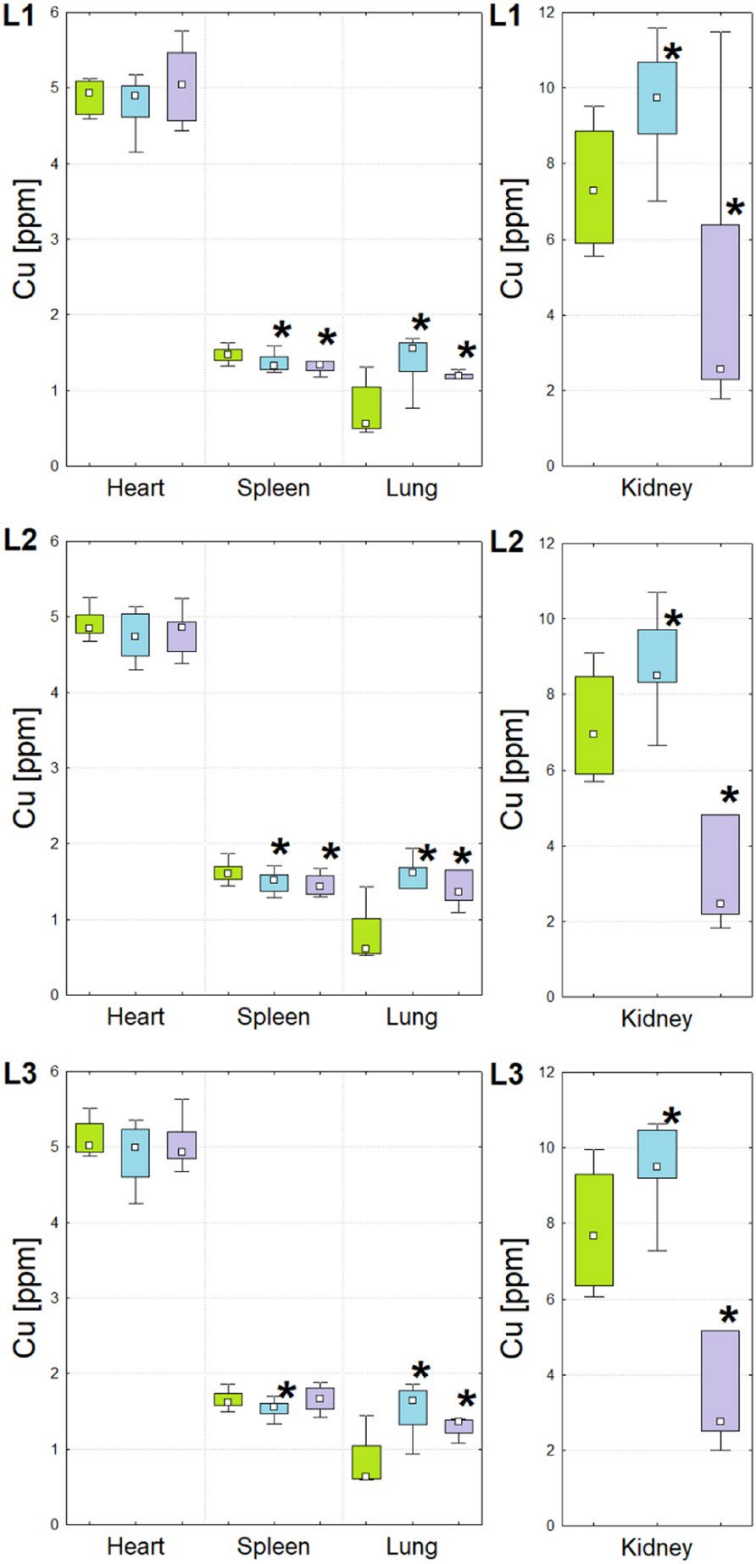
Box-and-whiskers plots presenting the ranges of Cu concentration in kidneys, hearts, lungs and spleens taken from animals representing N (green), T (blue) and U (violet) groups obtained through the cooperating laboratories. Median, interquartile range and minimal-maximal values are marked as a small square, a box and whiskers, respectively. The statistically significant differences (*p*-value < 0.05), comparing to control group, are signed with stars. For better visualization the data obtained for kidneys were presented separately.

**Figure 5:**
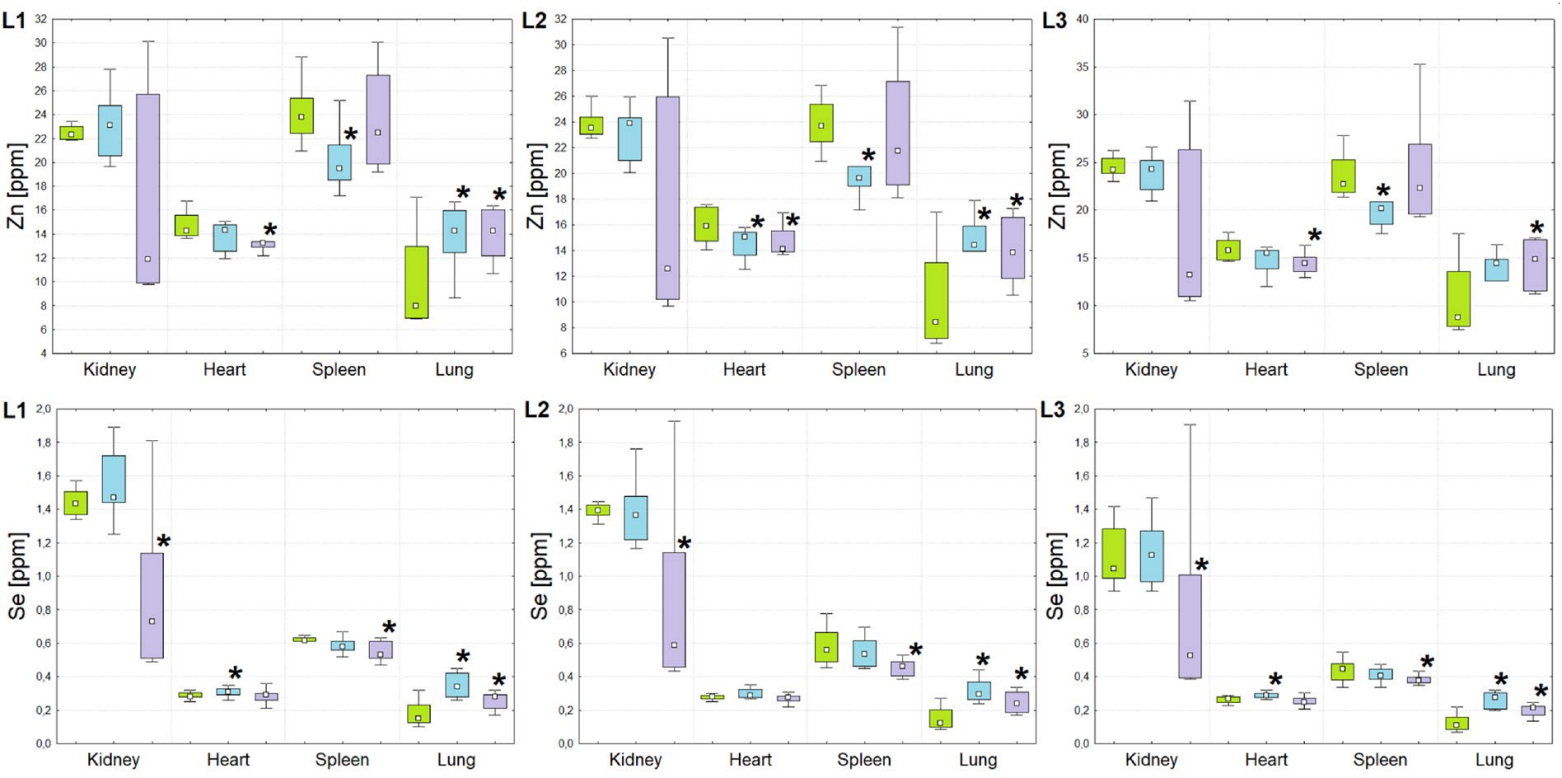
Box-and-whiskers plots presenting the ranges of Zn and Se concentrations in kidneys, hearts, spleens and lungs taken from animals representing N (green), T (blue) and U (violet) groups obtained through the cooperating laboratories (from left to right: L1, L2 and L3). Median, interquartile range and minimal-maximal values are marked as a small square, a box and whiskers, respectively. The statistically significant differences (*p*-value < 0.05), comparing to control group, are signed with stars.

As a result of the element analysis, for each of the examined 15 animals (5 animals × 3 study groups), the information on concentrations of P, S, K, Ca, Fe, Cu, Zn and Se in the 4 examined organs (kidney, heart, spleen and lung) was obtained. The content of elements was measured, independently, through three cooperating laboratories with the use of various apparatus and/or measurement conditions. Therefore, the statistical analysis of the obtained data, aiming at the evaluation of the significance of the differences between examined animal groups, was also done separately for them. In the Figs. 1, 2, 3, 4 and 5 the dispersions of the element concentrations in the examined organs for the three analyzed rat groups, that were obtained by the three collaborating laboratories (L1, L2 and L3), were compared. The data obtained for rats subjected to the implantation of T98 and U87MG cells were marked in blue and violet, respectively. In turn, the concentrations recorded for the control group were signed in green.

In order to better visualize the differences in the element composition occurring between the experimental and control rats, as well as the possible discrepancies in this range observed between the cooperating laboratories, the data presented in the Figs. 1, 2, 3, 4 and 5 are, additionally, summarized in the Table 1. Statistically significant differences comparing to normal group of animals are signed there as double arrows. The concentrations higher and lower in comparison to the normal organs are differentiated by the arrow direction.

**Table 1:**
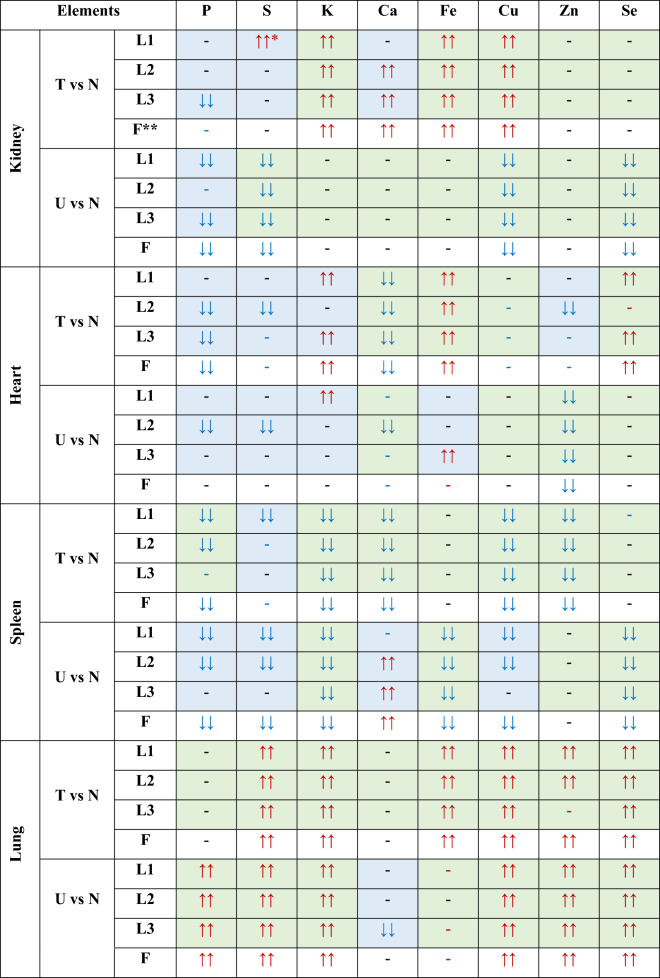
Statistically significant differences (Mann–Whitney *U* test, significance level of 5%) determined between experimental animals (groups T and U implanted with T98G and U87MG cells) and normal rats in cooperating laboratories (L1, L2, L3) together with the final agreed result (F).

*Double down/up arrows indicate statistically significant (*p*-value < 0.05) decrease (blue arrows)/increase (red arrows) of the concentration of element comparing to control rats;

**Observed differences in elemental composition were divided into fully (table cell marked in green) and partially compliant (table cell marked in blue);

***Final result determined based on the compliance of the results from at least two of participating laboratories.

Based on the results presented in the Table 1, the observed differences in elemental composition were classified as fully or partially compliant. When the same relation (or its lack) was found in the three cooperating laboratories, the result was treated as fully compliant. When the accordance was confirmed for two of three laboratories, the result was classified as partially compliant.

As one can see from the Figs. 1, 2, 3, 4 and 5 and from the Table 1, although in the three cooperating laboratories the samples were measured using various TXRF spectrometers and/or at different experimental conditions, the detected changes in elemental composition were mostly in a very good agreement. In case of elements with higher atomic numbers (Fe, Cu, Zn and Se), the 88% of the results were classified as fully compliant. The greatest number of discrepancies between the laboratories was found for P, S, K and Ca. The values of validation parameters including the limits of detection, intra- and inter-day precisions and trueness (Table S1 of the Appendix) determined for these elements point at the poorer accuracy and repeatability of the method in comparison with the heavier elements. This, in turn, influence the inter-laboratory precision of their analysis in the tissue samples. The mentioned problem was in details discussed in our earlier paper concerning the TXRF round-robin test for mammalian tissue samples^34^. However, it is necessary to mention that also in case of the elements with lower atomic numbers, more than 50% of the results were fully compliant and all the rest fulfilled the requirements of partial agreement.

In the further part of this paper we focused only on these element abnormalities (appearing after tumor cells implantation) that were observed in at least two of the cooperating laboratories, meaning that they have at least fulfilled the criterion of partial compliance. The final differences taken into consideration are mentioned in the rows F of the Table 1.

For both experimental groups significant elemental anomalies were observed in all examined organs after GBM cells implantation to the brain. According to the Figs. 1, 2, 3, 4 and 5 and Table 1, the most of elemental changes for both animal models were noted in the spleen and lungs. With one exception (the level of Ca in the spleen), the concentrations of analyzed elements in the spleen, measured for rats subjected to glioma cells implantation, were lower than in the control group. The opposite relationship was found for the lungs.

As it was shown in our earlier work^35^ and one can see in the Figures S1-S3 from the Appendix, after the implantation of T98G cells in the rat brain, no neoplastic tumor had developed. Despite this fact, in this study the animals from group T showed a number of elemental anomalies in the examined organs. Moreover, for the heart, for example, their number significantly exceeded the number of abnormalities observed in group U, for which massive tumors after implantation of U87MG cells had appeared (Fig. S3). Depending on the examined organ, the direction of the recorded elemental changes was the same for both models (decreased content of most elements in the spleen, increased in the lungs) or opposite, as for example in the kidneys.

The lower, comparing to controls, P levels were recorded in kidneys (group U), hearts (group T) and spleens (both experimental groups). In turn, in the lungs of animals implanted with U87MG cells, the concentration of this element increased. The concentration of S was changing mainly for group U—it decreased in the kidneys and spleen and increased in the lungs. The last regularity was also noted for animals implanted with cells of T98G line.

In animals from group T, the abnormalities in the range of K concentration were found for all examined organs, with the level of the element being elevated in kidneys, hearts and lungs, and decreased in the spleen compared to the control group. In case of animals subjected to implantation of the U87MG cell line, analogous abnormalities were noted, but only for spleens and lungs. Also the level of Ca changed more often for animals representing the T group than U. The rats subjected to T98G cells implantation showed a reduced level of this element in the spleen and heart and an increased in the kidneys, whilst these from the second experimental group were characterized only by the higher content of Ca in the spleen.

Anomalies in Fe accumulation were found mostly in case of T group, for which the increased level of the element was observed in kidneys, hearts and lungs. For both experimental groups the level of Cu was elevated in lungs but diminished in spleens. In turn, in kidneys the observed abnormalities were different for the two groups of animals subjected to glioma cells implantation, namely Cu content increased for T and decreased for U group. Both Zn and Se were elevated in lungs of experimental rats. The first from the mentioned elements was, additionally, decreased in hearts for U group and spleen of T group. In turn, Se was diminished in kidneys and spleens of rats subjected to U87MG cells implantation and increased in hearts of those that obtained T98G cells.

## Discussion and conclusions

The cancerogenesis process occurring in the brain may influence the response of the immune system also in other parts of the body and it may manifest itself with the changes in the elemental composition of tissues^36–38^. The elemental anomalies of various tissues and body organs associated with the occurrence of neoplastic processes are still at the stage of discovery and characterization^29–33^. Therefore, the aim of this investigation was identification of element abnormalities that appear in far distant organs as a result of implantation of human GBM cells into rat brain and/or subsequent tumor development. To achieve this goal, the kidneys, hearts, spleens and lungs were taken from animals subjected to implantation of T98G (described in literature as non-tumorigenic) and U87MG glioma cells (known as tumorigenic) and control rats^39–41^. The digested samples of organs were then measured in three European laboratories equipped with different commercially available TXRF spectrometers. The concentrations of elements, including P, S, K, Ca, Fe, Cu, Zn and Se, were determined using internal standard method and Ga was used for this purpose. To evaluate statistical significance of differences between experimental animals and normal rats, non-parametric Mann–Whitney *U* test was applied^42^ independently in each laboratory participating in the investigation. The following discussion and conclusions, based on the literature data obtained for samples of human and animal origin, relate to the results agreed upon for the three laboratories participating in the comparative study.

Phosphorus, as building element of nucleic acids, phospholipids, phosphoproteins or ATP, is involved in numerous processes occurring in cells and tissues^43–45^. Hubersch et al*.*, Srivastava et al*.* and Planeta et al*.* shown that cancerous GBM tissue is characterized by diminished concentration of this element, which is probably related to the decrease of phospholipids (i.e. lecithin, sphingomyelin) level within tumor^35,46,47^. The present study showed decreased concentration of P in most of the examined organs. The exception from this rule was elevated level of the element found in the lungs of animals from the U group. Decreased content of P observed in various organs of experimental animals may be an effect of hypophosphatemia, which, in turn, could result from kidneys dysfunction^48^. Such a conclusion seems to be supported by large element imbalance observed for this organ, especially in animals subjected to T98G cells implantation.

Fibroblast growth factor 23 (FGF23) is a protein and member of the fibroblast growth factor family, which participates in the regulation of phosphate level in plasma and the metabolism of vitamin D. It decreases reabsorption of phosphates in kidneys, allowing their excretion with urine. As it was shown by Bollenbecker et al*.*, FGF23 may be elevated during the systemic inflammation^49^. What is more, the higher level of FGF23 in lungs may lead to the elevated phosphate concentration within the organ^50,51^ and probably this phenomenon is responsible for significantly higher P level found for lungs taken from animals representing U group. It is necessary to mention that similar relation, but in this case not statistically relevant, was observed also for rats from T group.

Ca metabolism is closely related to the one of P. The linked homeostasis of both elements is crucial for proper functionality of neuromuscular system or mineralization process^52–56^. There is also an evidence for Ca playing an important role in cell signaling during the process of proliferation, and the Ca channels and Ca-regulated proteins show diverse and interconnected roles in the shaping of GBM biology and promotion of tumor growth^57^. The depletion of calcium ions level in the endoplasmic reticulum of glioma cells triggers their influx across the plasma membrane from intracellular space^57–60^. On a macro-scale, it causes imbalances in Ca level and the occurrence of the cell competition mechanism^57^. The elevated level of Ca in serum, called hypercalcemia, may occur in patients with neural tumors. However, it is described to be associated rather with astrocytic tumors than gliomas^57–60^. Our results showed relevantly decreased concentration of Ca in hearts of animals subjected to T98G cells implantation, and analogical trend for those implanted with U87MG cells. According to Shah et al*.*, the hypocalcemia may be caused by low Ca concentration in serum, which translates into the diminished level of the element in heart^61^. This in turn, may alter the flow of calcium through the voltage-gated cardiac calcium channels and lead to cardiac diseases^60–62^.

Sulphur is one of the most abundant mineral in the human body and its presence is related to the sulphur-containing amino acids—methionine, cysteine, cystine, homocysteine, homocystine, and taurine^63,64^. The occurrence and progress of GBM within brain is connected with the activity of γ-cystathionase and iron-sulphur centres of redox proteins^65^. M. Wróbel et al*.* observed the accumulation of sulphane sulphur in human gliomas and points to its importance for malignant cells proliferation and tumour growth. They linked this process with the diminished activity of γ-cystathionase. They showed also the correlation between the amounts of sulphane sulphur and the stage of the malignancy. High level of sulphane sulphur and a high GSH/GSSG ratio could result in elevated levels of hydrogen sulphide, that is often connected with the increase in malignancy of tumors^65^.

Iron-sulphur centers of redox proteins, consisting of Fe–S clusters, mediate electron transfer in the mitochondrial respiratory complexes. They are vital for the production and consumption of the energy in cells and are involved in the generation of reactive oxygen species (ROS)^66,67^. The organ affected by the cancerous process is experiencing the so-called Warburg effect—even in the presence of oxygen and properly functioning mitochondria, the glucose uptake radically increases in cells and lactate is produced^66–69^. The neoplasm present in the body states a great challenge and load for the immune system. Its response to any infection or an abnormality appearing in an organism is connected with the cytokines release. Their role is to alarm and affect the growth, proliferation and stimulation of cells involved in the immune response and also haemopoietic cells^68^. The cytokines activity, great energy requirements and altered energy metabolism in the neoplastic regions cause that the rest of the organism works at a minimum level necessary to maintain only the main of its functions. This reduced requirement and use of energy by remaining organs may be the reason of the diminished sulphur level observed in them^68,70^. The increased sulphur concentration in lungs of experimental rats may be, in turn, assigned to the pulmonary surfactant lining the alveoli. Han et al*.* pointed at its importance to the prevention of the dissemination and elimination of pathogens as well as modulating of the immune responses^71^. The overproduction of surfactant occurred in animals subjected to glioma cells implantation, probably, in the response to the ongoing inflammation in their respiratory system^71–73^.

Potassium is one of the most important electrolytes in the human body, as it is involved in maintaining the integrity of the skeleton, regulation of muscle contraction, blood pressure and nerve transmission. It is essential for the proper activity of all cells^74,75^. Hyperkalemia is a common disorder in patients suffering from cancers. The source of modifications of potassium channels and transmembrane proteins that mediate potassium within cells, may be different for various patients and may include renal failure, decreased potassium secretion and enhanced chloride reabsorption^76–79^. Low potassium in the spleen detected in our study may be related to the anemia caused by tumors progression. Despite the appropriate level of potassium in erythrocytes, the lower number of red blood cells undergoing lysis in the spleen may translate into the reduced level of the element within the organ^74,78,79^.

Iron is an essential metal in the human body. It is present in hemoglobin, myoglobin and various enzymes. The same, it takes part in oxygen transport, its storage in muscles, as well as energy production and regulation of diverse cell functions, including proliferation^80–82^. As mentioned above, it is also a building element of Fe-S centers of redox proteins mediating electron transfer in the mitochondrial respiratory complexes^66,67,82^. The high iron content detected in kidneys, hearts and lungs of animals representing T group may be associated with the altered iron metabolism and increased blood ferritin level connected with the process of tumorigenesis^83–85^. The mentioned above increased iron level was not observed in organs taken from animals subjected to the implantation of the most invasive glioma cell line U87MG. In rats from U group, the development of massive tumor within the brain was observed and their very poor health conditions made it necessary to shorten the experimental time. These animals presented the lowered iron content in the spleen, probably related to the anemia which is frequently observed in patients suffering from GBM. The anemia in GBM patients may be associated both with a reduced number of reticulocytes and a lowered iron-binding capacity. Therefore, the diminished concentration of iron in the spleen of rats from U group, was probably an effect of the reduced number of low-iron erythrocytes hemolysis in this organ^84^.

In human body, copper is required for the proper action of enzymes involved in aerobic metabolism, such as cytochrome c oxidase in the mitochondria, dopamine monooxygenase in the brain, lysyl oxidase in connective tissue and ceruloplasmin^86^. Together with iron, this element takes part in the formation of red blood cells. It was also proven that the growth and metabolism of cancer cells, due to the elevated angiogenesis, require more copper^87–90^. As copper is also strictly associated with the progress of inflammation process, including the cytokines production, during inflammation increased levels of the element are observed in serum^87^. Probably, just ongoing in kidneys and lungs inflammation process caused the increase of the copper level in these organs in case of animals subjected to T98G cells implantation. For the U group the mentioned effect was noticed only for lungs. In turn, both experimental groups were characterized by decreased copper concentration in spleen, which at least in case of U group, may be explained by anemia.

The role and activity of Zn in the body are tightly bound to those of Fe and Cu. Zn is crucial for the development and function of immune cells, hematopoiesis, cell signaling and the inflammatory response. It also participates in DNA synthesis and protein production^91,92^. Mehrian-Shai et al*.* found that the protein p53, which has a suppressive influence on cancers, is activated by zinc^93^. Thus, the detected zinc deficiency could be associated with the modulation of immune system activity by the developing GBM. In this context, an anemia of chronic disease and the problems with nutrients absorbtion should also be taken into account^92–95^. In turn, the increased level of zinc detected in lungs of animals representing both experimental groups may point at the occurrence of the organ oxidative stress, but at those stage of investigation the results are inconclusive^66,96^.

Selenium is a microelement important for human body from the point of view of the antioxidant processes and their role in the immune system response. Its presence in proteins is connected with the amino acid of selenocysteine^97^. Zhang et al*.* noticed that the level of the element is higher within in the region of brain tumor comparing to the healthy tissue^98^. Similar observations were done during our previous study, analyzing brain samples taken from the area of glioma cells implantation^35^. The increased demand and accumulation of selenium in GBM tissue may be the cause of its low serum level. In turn, the mentioned effect, together with the anemia of chronic disease, may explain the detected depletion of selenium in the kidneys and spleen of rats from U group^97–99^. On the other hand, an elevated content of the element in lungs, in case of both experimental groups, and in heart of animals from T group, could be associated with the selenium-driven enhancement of cell-mediated and humoral immunity^97–100^. The increased content of selenium in heart may be also explained by the production of antioxidative agents, containing i.e. selenium, in the answer of ROS travelling through the coronary system to regions highly supplied with blood^99–102^.

Summarizing, the conducted studies have shown that the implantation of human glioma cells into the rat brain is associated with a number of elemental anomalies in distant organs. These changes occur even when there is no tumor developing in the brain. The observed disorders of element homeostasis may result from many processes occurring in the body as a result of implantation of cancer cells or the development of GBM, including inflammation, anemia of chronic disease or changes in iron metabolism. The evaluation of the anomalies detected in the 3 laboratories participating in the inter-comparison study showed a good agreement between obtained results. When all the experimental data were taken into account, the full compliance of the results was observed for 72% of cases. In case of elements with higher Z, it was better, as the full compliance of the results was found for 88% of cases.

## Materials and methods

### Experimental animals

The animals used in this study were male Wistar rats originating from the husbandry of the Department of Experimental Neuropathology of the Institute of Zoology and Biomedical Research, Jagiellonian University in Krakow. All animal-use procedures were approved by 2nd Local Institutional Animal Care and Use Committee in Krakow (agreement no. 119/2016) and were executed in consonance with relevant guidelines and regulations. All methods are reported in the paper in accordance with ARRIVE guidelines (https://arriveguidelines.org).

### Glioma cells implantation

At 9 weeks of age, the rats were divided into 3 groups: N, T and U. Each group consisted of 5 individuals. N group included naive control animals, while the rats from groups T and U were subjected to the implantation of glioma cells into the brain. T98G and U87MG cell lines from ATCC were used for this purpose, respectively. The day before and on the day of surgery, the animals were weighed and cyclosporine was given them intravenously (Sandimmun 50 mg/mL, Novartis Poland) at a dose of 10 mg/kg of body mass for immunosuppressive purposes. Before implantation, the rats were anesthetized in an isoflurane-filled desiccator (Aerrane, Baxter Poland) and throughout the whole surgery the same substance was administered to them through inhalation. The implantation site was determined stereotaxically in the left hemisphere (coordinates antero-posterior: − 0.30 mm; medio-lateral: 3.0 mm; dorso-ventral: 5.0 mm, Paxinos and Watson 1986). Cell suspension in a volume of 5 μL was introduced to the brain through an intracranial hole drilled beforehand. The concentration of T98G and U87MG cells in the suspension was 50,000 and 5000 cells/μl, respectively. After glioma cells injection, the wound was sutured with a stapler and disinfected. After surgery, the animals were administered cyclosporine (Novartis Poland) at a daily dose of 5 mg/kg of body mass. Furthermore, for the first 7 days after glioma cells implantation, the rats were given an antibiotic (Sul-Tridin 24%, ScanVet, Poland). Experiment, from the day of implantation, lasted 21 days for groups N and T. In the case of the U group, due to the very poor animal health, the experiment was terminated 15 days after the surgery. Rats were sacrificed by intravenous Euthasol-Vet administration (Euthasol vet 400 mg/mL, Le Vet B.V.) at doses appropriate for their weight. From each rat the kidney, heart, spleen and lung were taken. The organs were weighed, frozen in liquid nitrogen and separately packed in sterile bags, which were stored in ultra-freezer until the mineralization procedure.

### Sample preparation

Microwave-assisted acid digestion was performed using the Speed Wave 4 system (Berghof). Each organ was placed in a separate Teflon vessel (DAP100) together with the high purity 65% nitric acid (Suprapur, Merck), the volume of which was typically 2.5 ml per 1g of tissue. The sample masses varied from approximately 300 mg for spleens to 2 g for livers. For the purpose of quantitative elemental analysis via the internal standard method, 100 μl of the 1000 ppm gallium solution (Gallium ICP standard in HNO_3_ 2–3% 1000 mg/l Ga Certipur^®^, Merck) was added to the entire volume of the digested sample. Afterwards, from such prepared solution, subsamples of 1 ml volume were taken to the separate vials and stored at a temperature of 5 °C until they were sent to the cooperating laboratories.

### Apparatus and experimental conditions

The measurements of digested organ samples were carried out in three laboratories involved in the ENFORCE TXRF COST Action 18130. These were X-ray Fluorescence Laboratory of the Faculty of Physics and Applied Computer Science at the AGH University of Science and Technology (Laboratory 1, Krakow, Poland), Laboratory of X-ray Methods of the Center for Research and Analysis at the Jan Kochanowski University (Laboratory 2, Kielce, Poland) and TXRF Laboratory of the Interdepartmental Research Service at the Autonomous University of Madrid (Laboratory 3, Madrid, Spain). The TXRF spectrometers used for investigation as well as experimental conditions used in particular laboratories engaged in cooperation are listed in the Table 2. The detailed description of preliminary and measurement procedures was presented elsewhere^34^.

**Table 2:** Characteristics of the equipment and experimental conditions used in cooperating laboratories.

Laboratory	Laboratory 1 (L1)	Laboratory 2 (L2)	Laboratory 3 (L3)
Spectrometer	Nanohunter II Rigaku	S2 PICOFOX Bruker Nano	S2 PICOFOX Bruker Nano
Tube anode	Mo	Mo	Mo
Tube voltage (kV)	50	50	50
Tube current (mA)	12	0.6	0.6
Glancing angle (°)	0.04	< 0.1	< 0.1
Acquisition time (s)	1000	1000	500
Deposited volume (μl)	6	6	5
Sample carrier	Super Frost (Menzel) glass slides	Quartz glass	Quartz glass

### Data analysis

The concentration of each element in the organ was calculated from the number of counts recorded for its peak in the sample spectrum, taking into account the concentration of the internal standard (Ga) in the sample, number of counts for Ga peak in the sample spectrum and the relative sensitivity of the TXRF system for the measured element. The details of calculations were presented elsewhere^34^.

For the evaluation of the statistical significance of the differences in elemental composition of examined organs between rats subjected to glioma cells implantation (T and U groups) and the control group non-parametric Mann–Whitney *U* test was utilized, and the appropriate calculations were done using Statistica software version 7.1. The statistical analysis was done independently for each laboratory participating in the study.

### Supplementary Information

Supplementary Information.

## Data Availability

The datasets analysed during the current study available from the corresponding author on reasonable request.
