# Supplementary material for: Glioblastoma multiforme influence on the elemental homeostasis of the distant organs: the results of inter-comparison study carried out with TXRF method
Source: Sci Rep. 2024 Jan 13;14:1254. doi: 10.1038/s41598-024-51731-2 (PMC10787745; doi:10.1038/s41598-024-51731-2)
Supplement: Supplementary file 1 — Supplementary Information. [file 41598_2024_51731_MOESM1_ESM.pdf]

Appendix

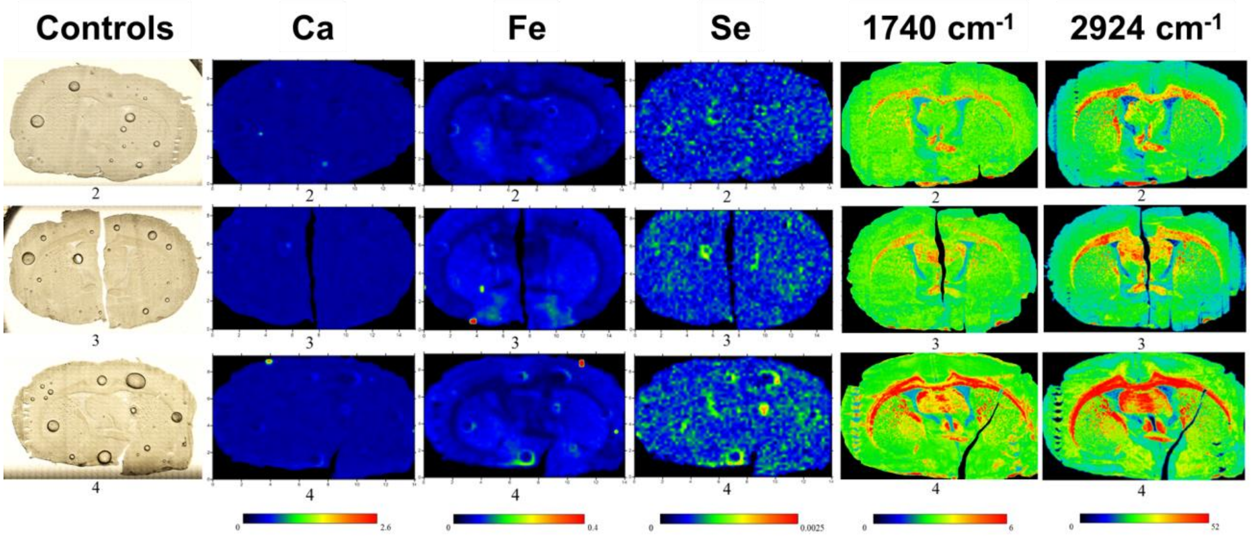

Figure S1. Histological images and chemical maps presenting the distributions of calcium, iron, selenium, the compounds containing carbonyl groups (absorption band at 1740 cm^{-1}) and the lipids (absorption band 2924 cm^{-1}) obtained for the slices of brain taken from three normal rats. The maps of element accumulations were obtained with the X-ray fluorescence microscopy whilst those showing the distributions of organic compounds with FTIR microspectroscopy.

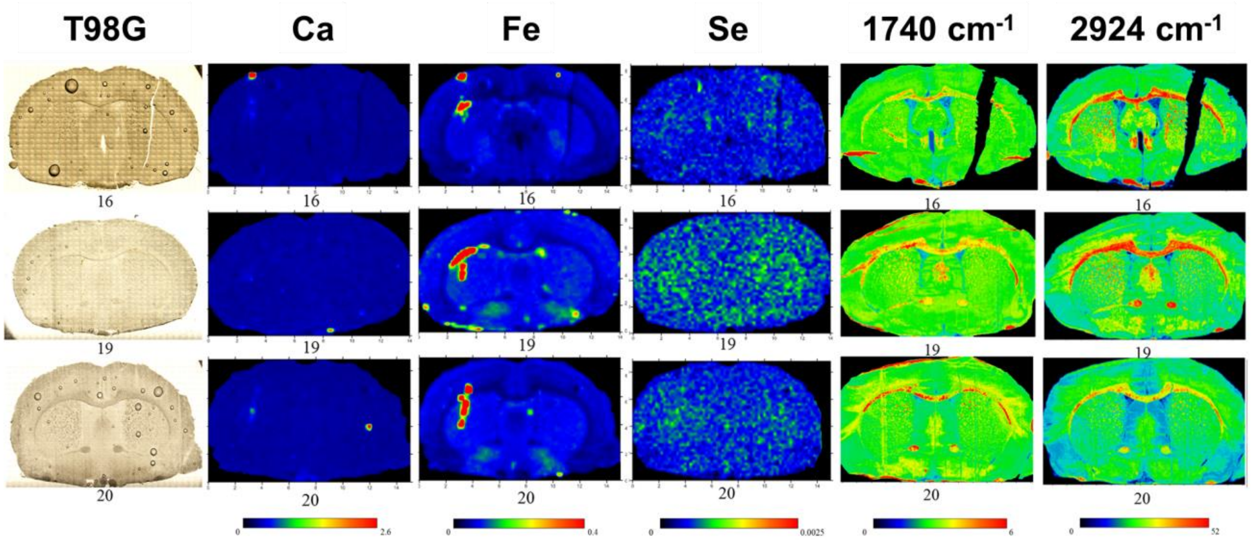

Figure S2. Histological images and chemical maps presenting the distributions of calcium, iron, selenium, the compounds containing carbonyl groups (absorption band at 1740 cm^{-1}) and the lipids (absorption band 2924 cm^{-1}) obtained for the slices of brain taken from the site of T98G cells implantation.

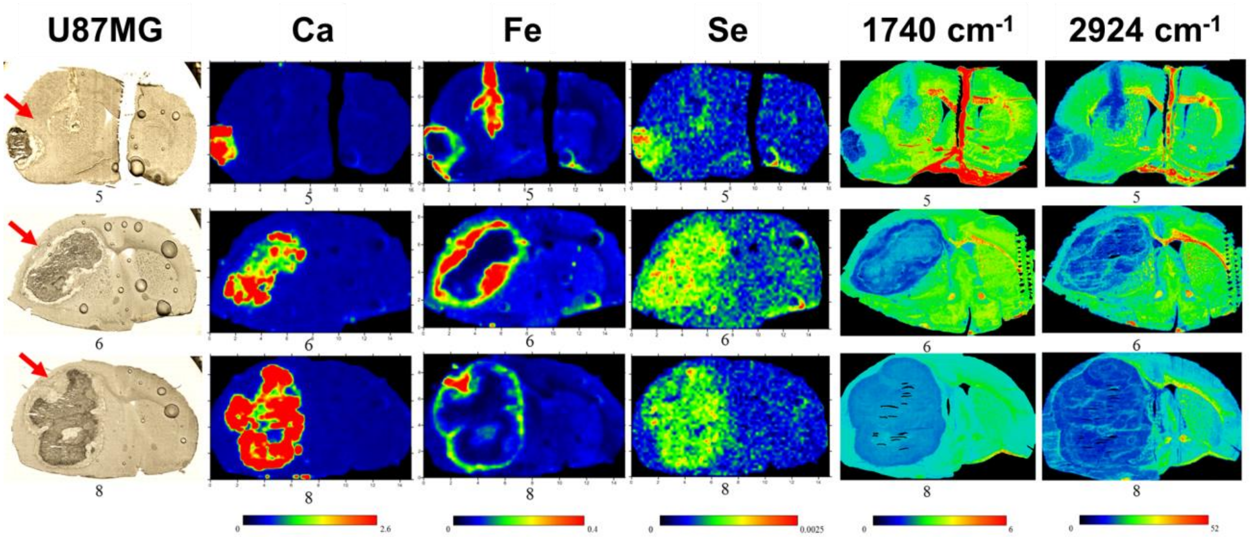

Figure S3. Histological images and chemical maps presenting the distributions of calcium, iron, selenium, the compounds containing carbonyl groups (absorption band at 1740 cm^{-1}) and the lipids (absorption band 2924 cm^{-1}) obtained for the slices of brain taken from the site of U87MG cells implantation.

Table S1. The values of validation parameters determined for the heart sample of normal rat in the 3 laboratories participating in the study.

Element	Laboratory	LOD [mg/kg]	Intra-day precision [%]*	Inter-day precision [%]	Trueness [%]**
**P**	**Lab1**	7.58	2.30	0.41	68
	**Lab2**	13.40	16.64	7.94	193
	**Lab3**	5.16	0.47	3.34	103
**S**	**Lab1**	4.63	3.64	1.88	89
	**Lab2**	3.89	17.38	8.18	121
	**Lab3**	2.51	0.62	0.86	111
**K**	**Lab1**	1.55	2.95	2.33	116
	**Lab2**	0.766	8.25	5.34	114
	**Lab3**	0.833	0.53	1.74	120
**Ca**	**Lab1**	0.980	12.11	16.11	154
	**Lab2**	0.374	13.46	11.85	112
	**Lab3**	0.433	0.56	2.18	121
**Fe**	**Lab1**	0.0677	0.64	0.91	96
	**Lab2**	0.0464	1.46	1.13	97
	**Lab3**	0.0817	0.38	1.05	99
**Cu**	**Lab1**	0.0417	2.14	1.38	97
	**Lab2**	0.0325	1.61	0.63	99
	**Lab3**	0.0493	1.58	1.24	107
**Zn**	**Lab1**	0.0538	5.79	0.31	99
	**Lab2**	0.0306	1.78	0.41	104
	**Lab3**	0.0457	0.42	0.80	104
**Se**	**Lab1**	0.0228	4.54	5.44	167
	**Lab2**	0.0151	5.75	1.29	151
	**Lab3**	0.0213	4.60	11.09	134

* Intra-day and inter-day precision was expressed as coefficient of variation;

** Trueness [%] was calculated according to the formula: (experimental value/certified value)*100. This was done based on the comparison of the results measured with TXRF and the reference method (ICP-MS for Se and ICP-OES for all remaining elements).
